# The *N*-Acetylglutamate Synthase Family: Structures, Function and Mechanisms

**DOI:** 10.3390/ijms160613004

**Published:** 2015-06-09

**Authors:** Dashuang Shi, Norma M. Allewell, Mendel Tuchman

**Affiliations:** 1 Center for Genetic Medicine Research and Department of Integrative Systems Biology, Children’s National Medical Center, the George Washington University, Washington, DC 20010, USA; E-Mail: mtuchman@childrensnational.org; 2 Department of Cell Biology and Molecular Genetics and Department of Chemistry and Biochemistry, College of Computer, Mathematical and Natural Sciences, University of Maryland, College Park, MD 20742, USA; E-Mail: allewell@umd.edu

**Keywords:** arginine biosynthesis, urea cycle, *N*-acetylglutamate synthase, crystal structures, catalysis and regulation

## Abstract

*N*-acetylglutamate synthase (NAGS) catalyzes the production of *N*-acetylglutamate (NAG) from acetyl-CoA and l-glutamate. In microorganisms and plants, the enzyme functions in the arginine biosynthetic pathway, while in mammals, its major role is to produce the essential co-factor of carbamoyl phosphate synthetase 1 (CPS1) in the urea cycle. Recent work has shown that several different genes encode enzymes that can catalyze NAG formation. A bifunctional enzyme was identified in certain bacteria, which catalyzes both NAGS and *N*-acetylglutamate kinase (NAGK) activities, the first two steps of the arginine biosynthetic pathway. Interestingly, these bifunctional enzymes have higher sequence similarity to vertebrate NAGS than those of the classical (mono-functional) bacterial NAGS. Solving the structures for both classical bacterial NAGS and bifunctional vertebrate-like NAGS/K has advanced our insight into the regulation and catalytic mechanisms of NAGS, and the evolutionary relationship between the two NAGS groups.

## 1. Introduction

*N*-acetylglutamate (NAG) is the initial precursor of arginine in the arginine biosynthetic pathway in microorganisms and plants, while the same compound is an essential cofactor of carbamoyl phosphate synthetase 1 (CPS1) in the urea cycle [1,2,3]. Acetylation of the early precursors of arginine prevents the spontaneous cyclization of the semialdehyde with the unprotected α-amino group of glutamate separating arginine biosynthesis from proline biosynthesis. The flow of the acetylated precursors of arginine often starts with NAG and ends with acetylornithine, identification of the novel *N*-acetylornithine transcarbamylase suggests that the acetylated precursors of arginine extend beyond acetylornithine in certain bacteria [4,5,6]. Deacetylation of acetylornithine to form ornithine is achieved by one of two enzymes, either acetylornithine deacetylase (acetylornithinase) in the linear pathway, or ornithine acetyltransferase in the cyclic pathway. The cyclic pathway observed in most bacteria and plants recycles the acetyl group to form NAG, which is energetically more economical and is considered traditionally to have evolved more recently [7]. The formation of arginine is catalyzed by three subsequent enzymes, ornithine transcarbamylase, argininosuccinate synthase, and argininosuccinate lyase, via citrulline and argininosuccinate.

Enzymes catalyzing the acetylation of l-glutamate exhibit a high degree of diversity compared to the other enzymes of arginine biosynthesis. The best known is the classical bacterial *N*-acetylglutamate synthase (NAGS), which was identified in *Escherichia coli* and *Pseudomonas aeruginosa* [8,9,10,11]. This enzyme is encoded by a single gene (*argA*), and consists of two domains. The N-terminal domain is similar to amino acid kinases (AAK), which are also found in the next enzyme of arginine biosynthesis, *N*-acetylglutamate kinase (NAGK). The C-terminal, *N*-acetyltransferase (NAT) domain is a member of the Gcn5-related acetyltransferase (GNAT) family that transfers the acetyl group from acetyl-CoA to various amino groups. The protein sequence of members of the GNAT family is highly diverse with overall similarities as low as 5%–10%. Even though human and rat NAGS activities have been known for more than thirty years [12,13,14], the mammalian *NAGS* genes were not identified until 2002, probably because of the low sequence similarities to bacterial homologues [15,16]. Interestingly, some bacterial NAGS-like sequences with higher sequence similarities to mammalian NAGS than to classical bacteria NAGS, were also identified in several *Xanthomonadales* and marine α-proteobacteria. Further studies have revealed that these proteins have both NAGS and NAGK activities, catalyzing the first two reactions of arginine biosynthesis as a bifunctional NAGS/K enzyme [17,18]. Phylogenetic analysis of NAGS sequences from bacteria, fungi, plants and vertebrates demonstrated that classical bacteria NAGS and plant NAGS belong to one group (defined as the bacteria-like NAGS group), while vertebrate and fungal NAGS and bifunctional NAGS/K cluster into another group (defined as vertebrate-like NAGS group), but with higher divergence within the group [17]. Interestingly, fungal NAGK also belongs to the vertebrate-like NAGS group, even though it does not have NAGS activity.

**Figure 1:**
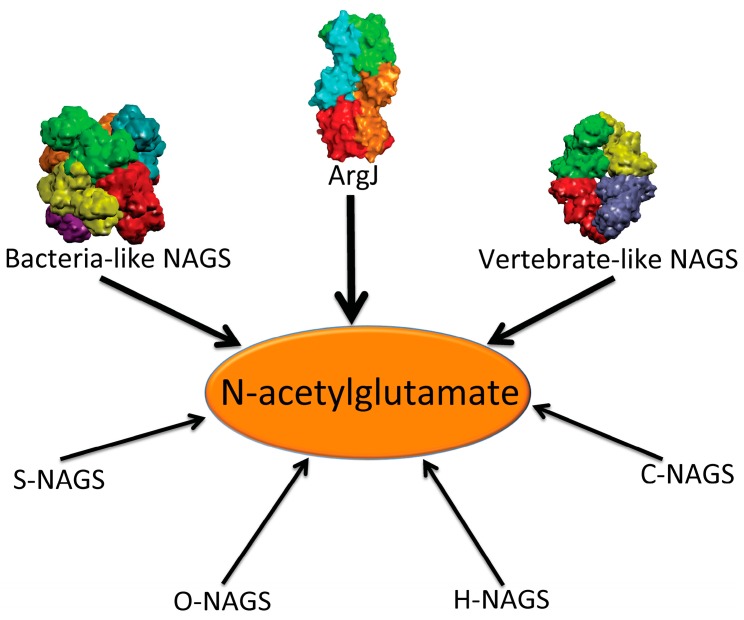
Enzymes that produce *N*-acetylglutamate. Bacteria-like *N*-acetylglutamate synthase (NAGS) include the classical bacterial NAGS and plant NAGS, which have hexameric architectures, represented by NAGS from *Neisseria gonorrhoeae* (PDB code 2R8V). Vertebrate-like NAGS include the bifunctional bacterial NAGS/K, mammalian NAGS, fungal NAGS and other vertebrate NAGS, which have tetrameric architectures, represented by the bifunctional NAGS/K from *Maricaulis maris* (PDB code 3S6G). These two groups of NAGS are typical NAGS enzymes in that they have both AAK and NAT domains and catalyze the formation of NAG from AcCoA and glutamate. They are the focus of this review. ArgJ is the mono- or bi-functional ornithine acetyltransferase that catalyzes the production of NAG from acetylornithine and glutamate (mono-) and AcCoA and glutamate (bi-). This enzyme is likely to be a heterotetramer, represented by argJ from *Mycobacterium tuberculosis* (PDB code 3IT4). Other atypical NAGSs exist in certain bacteria but no structures have been determined so far. The short version of NAGS (S-NAGS), for example argA from *M. tuberculosis* has only NAT domain. The NAGS encoded by the *argO* gene of *Campylobacter jejuni* (O-NAGS) consists of 146 amino acids, which broadly relate to the GNAT family. H-NAGS has been identified in *Moritella abyss* and *Moritella profunda* with argH fused to the N-terminal end of the NAT related domain. C-NAGS is a novel type of NAGS, recently identified in *Corynebacterium glutamicum*, whose sequence does not have any relationship to other known NAGS.

In addition to the above enzymes, which have AAK and NAT domains, several other proteins are able to catalyze the formation of NAG (Figure 1). The best-known is *argJ* encoded ornithine acetyltransferase (OAT), which catalyzes the reversible acetyl exchange between ornithine and glutamate. OATs have been characterized *in vitro* or *in vivo* as mono- or bifunctional enzymes [19,20]. The bifunctional OAT catalyzes both the acetyl transfer reaction from acetylornithine to glutamate and from acetyl-CoA to glutamate as does the typical NAGS. No detectable sequence similarity is observed between bifunctional OATs and typical NAGS. It seems that the CoA moiety of acetyl-CoA does not enter the catalytic site of OAT [19], in contrast to the typical NAGS, which recognizes CoA [21]. Mature active OAT derives from a preprotein, which undergoes autocatalytic cleavage to form a biologically active hetero tetramer [22,23].

Synthesis of NAG can also be catalyzed by other proteins including *argO* encoded NAGS (O-NAGS) first identified in *Campylobacter jejuni*, S-NAGS, a short version of NAGS first identified in *M. tuberculosis* [24], and argH fused NAGS (H-NAGS) [7,25]. O-NAGS has only 146 amino acids [26] and although its sequence is related to the GNAT family, it does not have significant similarity to the NAT domain of the typical NAGS. S-NAGS is able to catalyze the formation of NAG, but has an extremely high *K*_m_ for glutamate [24]. H-NAGS, which includes a short version of NAGS fused to the C-terminus of argininosuccinate lyase (argH), has been found in some bacteria in the Alteromonas-Vibro group and is able to complement an *argA E. coli* mutant [27]. These enzymes have been reviewed in detail by Xu *et al.* [7]. More recently, a novel type of NAGS, the sequence of which is not related to any known NAGS, was identified in *Corynebacterium glutamicum* [25]. The variety of these enzymes raised the question of how and why proteins with such high divergence have the ability to catalyze the formation of the same compound, NAG.

The acetylation of the α-amino group of glutamate was once thought to be the only way to prevent spontaneous cyclization of unprotected intermediates in the arginine biosynthetic pathway. The discovery of the novel *N*-succinyl-l-ornithine transcarbamylase indicates that different organisms may use different mechanisms to protect the α-amino group of glutamate [28,29]. Although the gene that encodes an enzyme that succinylates glutamate has not been identified, the identification of a GNAT-like succinyltransferase suggests that a similar enzyme will catalyze the succinylation reaction of glutamate [30]. Yet another mechanism for protecting the α-amino group of glutamate was identified when a small protein, LysW, was shown to also protect the α-amino group of glutamate by covalently linking the amino group of glutamate to the γ-carboxyl group of the terminal glutamate of LysW [31]. A RimK-like protein [32] encoded by *ArgX* (a homolog of *LysX*), with no sequence similarity to any known NAGS, catalyzes the above reaction. Notably, this mechanism was discovered in a member of the archaea, which use the same set of enzymes for l-lysine and l-arginine biosynthesis.

This review will focus on the function, structures and mechanisms of the typical NAGS, which have been well characterized. Fifteen structures of these enzymes have been determined with seven in the bacteria-like NAGS group (PDB codes: 4I49, 3D2M, 3D2P, 2R8V, 2R98, 3B8G and 3E0K) and eight in the vertebrate-like NAGS group (4NEX, 4NF1, 4KZT, 4K30, 3S6H, 3S6G, 3S6K and 3S7Y). They include full-length and sub-domain structures, complexed with inhibitors as well as substrates. No NAGS structure from any fungi or plant has been determined until now, probably because of instability of most NAGS from other organisms. The structural information gleaned so far offers new insights into structure-function relationships as well as the catalytic and regulatory mechanisms.

## 2. Early Biochemical Characterization of NAGS

NAGS from *E. coli* (ecNAGS) is the best characterized member of the bacteria-like NAGS group. The protein has been purified to homogeneity and its biological and catalytic properties have been reported [8,9]. The enzyme seems to shift between trimeric and hexameric states depending on its concentration and the presence of ligands [8]. ecNAGS shows a relatively high substrate specificity towards l-glutamate and acetyl-CoA. Among the other amino acids tested, only l-glutamine and l-2-aminoadipate have acetylation activity of 6.8% and 1.3% of that of l-glutamate, respectively. Among the l-glutamate acyl donors that were tested, propionyl-CoA was the only other effective donor with 4.5% of the acetyl-CoA acylation rate [8].

The activity of ecNAGS is inhibited by l-arginine, with 50% activity reduction at 2 mM. ecNAGS is also inhibited, but to a lesser degree, by the reaction products NAG and CoA, with 50% inhibition at 25 and 2.5 mM, respectively. Feedback inhibition by l-arginine was enhanced additively by CoA and NAG, suggesting that l-arginine binds to the enzyme at a different site. Stabilization of the hexameric form of NAGS by NAG and l-arginine indicates that the regulation of the enzyme probably involves ligand-induced changes of its oligomerization state.

Mammalian NAGS is found in the mitochondrial matrix of cells of the liver and intestines but not the kidney or spleen [33,34], and has been purified to apparent homogeneity from rat and human liver [12,13]. The molecular weight of the human holoenzyme is 190,000. Human liver NAGS affinity constants (*K*_m_) were reported as 4.4 mM for acetyl-CoA and 8.1 mM for l-glutamate [12], quite different from the values of 0.7 and 1 mM, respectively, for the rat enzyme [13]. The later numbers are in better agreement with recent biochemical studies on the recombinant enzymes [35].

Mammalian NAGS acetylates almost exclusively l-glutamate, with little activity with glutamine (5.0%) and glycine (2.9%) and no acetylation of other amino acids (<1.0%). l-glutamate-hydroxamate shows low activity as an acyl acceptor (15.5%), whereas no activity was found for l-glutamate-monoethyl ester and -hydrazide. Glutamate analogues that have longer carbon chains, such as dl-aminoadipate (5.2%) and dl-aminopimelate (4.0%), show low activity. The acetyl-CoA substrate is also very specific, showing low activity with propionyl-CoA (4.3%) and no activity with other acyl-CoA derivatives [13].

Enzymatic activity in the presence of saturating concentrations of substrates more than doubles (2–5-fold) in the presence of l-arginine with an activation constant (*K*_a_) of 30–50 µM *in vitro*. Since no other amino acid has this activity and since the *K*_a_ is in the range of liver arginine concentrations *in vivo* [12,13,14,34,36], it is likely that arginine has a role in regulating NAG production. Arginine seems to increase the enzyme velocity without an effect on affinity for the substrates [37]. It is transported into mitochondria and therefore could activate NAGS *in vivo* [38]. The activation of NAGS by arginine seems to be very specific, since among the structural analogues of arginine, intermediates of the urea cycle, and amino acids, only l-argininic acid produces a similar activation [13]. Activation by argininic acid and arginine are not additive, implying that they probably bind to the same site. NAGS can also be activated by cationic polypeptides such as protamines, polyarginine and polylysine, but the activation is additive relative to that of arginine [13]. Mammalian NAGS appears to be a trimer but its oligomerization state seems to depend on the concentration of l-arginine [39]. These cationic polypeptide activators seem to stabilize the conformation of activated NAGS. A better understanding of arginine’s effect on NAGS activity could clarify its role in NAGS activation.

## 3. Recent Biochemical Characterization of Recombinant NAGS

Recently, Rubio’s group revisited the classical NAGS from *Pseudomonas aeruginosa* (paNAGS) using recombinant protein and mutagenesis techniques [10,39,40,41]. Gel filtration assays revealed that paNAGS exists in a non-dissociating hexamer regardless of the presence and absence of arginine. Mutation studies demonstrated that mutations of the site at which arginine is bound, specifically the C-terminal end of the AAK domain, did little to change the substrates’ kinetic parameters. The AAK and NAT domains can be expressed separately. The NAT domain has NAGS activity and is arginine insensitive even though the concentration of glutamate required for activity with the NAT domain alone was 25-fold higher than for full-length NAGS. Gel filtration experiments demonstrated that the AAK domain alone assembled as a hexamer, implying that the hexameric architecture of the full-length NAGS is nucleated by a hexamer ring of AAK domains that closely resembles the arginine sensitive bacterial NAGK [42]. In contrast, the NAT domain probably exists as a monomer, although this needs to be further confirmed by additional experiments. Mutagenesis studies further demonstrated that the linker amino acids between the two domains are important for regulation of NAGS activity by arginine [41].

Because of the low sequence similarity between mammalian NAGS and classic bacterial NAGS, the gene that encodes mammalian NAGS was not identified until 2002 [15,16,43]. In addition to the AAK and NAT domains, mammalian NAGS has 40–50 additional amino acids at its N-terminus, termed the “variable domain” because of its low sequence similarity even among mammalian NAGS. The biochemical properties of mature NAGS (with variable domain but without mitochondrial target signal) and conserved NAGS (without variable domain and mitochondrial target signal) recombinant human and mouse proteins were compared [35]. The presence of 1 mM arginine enhances NAGS activity about twofold. More interestingly, conserved NAGS (without the variable domain) has twofold higher activity than mature NAGS (with the variable domain), in the presence of arginine. The variable domain is rich in charged amino acids and prolines, and likely devoid of secondary structure. Such unstructured domains are often important for protein–protein interactions. Whether and how the variable domain is involved in regulation of NAGS activity or interaction with other enzymes such as CPS1 needs to be further elucidated.

## 4. Overview of the Structural Fold

**Figure 2:**
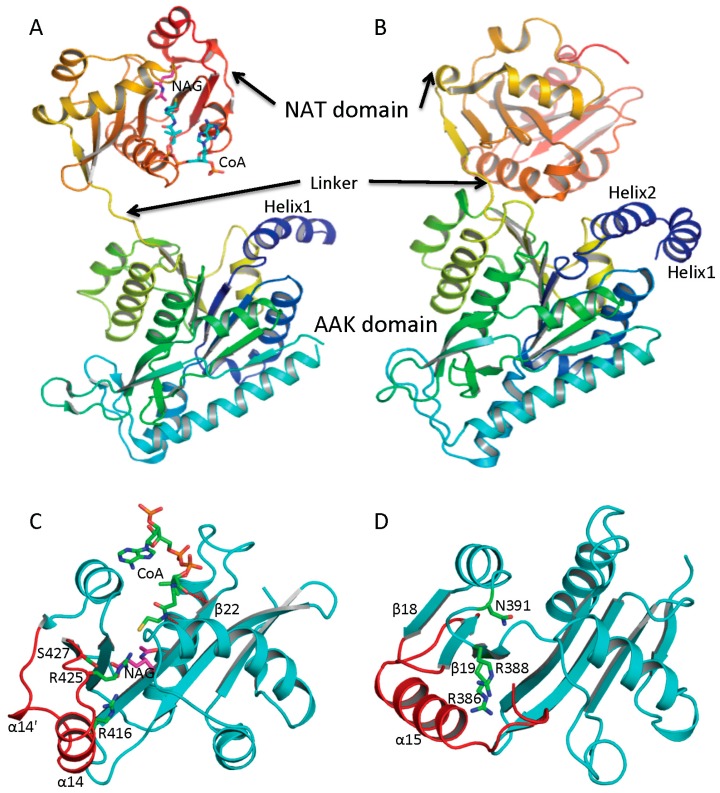
Ribbon diagram of subunit structures of ngNAGS and mmNAGS/K. (**A**) Subunit structure of ngNAGS in the absence of arginine. The structure consists of two domains, amino acid kinase (AAK) domain and *N*-acetyltransferase (NAT) domain, linked together by a flexible linker. No domain-domain interactions are observed within the subunit except via the linker; (**B**) Subunit structure of mmNAGS/K in the absence of arginine. The linker between AAK and NAT domains is shorter than that in ngNAGS. Some domain-domain interactions are observed. The structures are colored as a rainbow gradually changed from dark-blue for the N-terminus to red for the C-terminus; (**C**) Ribbon diagram of the NAT domain of ngNAGS. The bound CoA and NAG are shown in green and magenta sticks, respectively. Three residues, R416, R425 and S427, which are involved in binding NAG, are shown as green sticks; and (**D**) Ribbon diagram of NAT domain of mmNAGS/K. The last helix, α15, occupies the equivalent position of α14 of ngNAGS. Three residues, R386, R388 and N391, which are potentially involved in binding NAG, are shown as green sticks. The major differences in the NAT domains between ngNAGS and mmNAGS/K are colored in red.

Determination of the crystal structures of NAGS from *Neisseria gonorrhoeae* (ngNAGS), which belongs to the classical bacteria-like NAGS group, and bifunctional NAGS/K structures from *Maricaulis maris* (mmNAGS/K) and *Xanthomonas campestrics* (xcNAGS/K), which belong to the vertebrate-like NAGS group including mammalian NAGS allows detailed structural characterization of each group possible. Even though there are significant sequence differences between the bacteria-like and vertebrate-like groups, both consist of two independent domains [44,45] (Figure 2A,B). Furthermore, the overall fold of each domain of these two groups of NAGS is very similar, although some differences exist. The N-terminal domain has typical the AAK domain with an eight-stranded β sheet as its central core and α helices hanging on both sides. The superimposition of AAK domains of mmNAGS and ngNAGS results in a root mean square deviation of 2.7 Å, with over 260 aligned residues. The most significant differences are identified in the N-terminal segment. The N-terminal segment of mmNAGS/K has two helices; in contrast, ngNAGS has only one as does arginine sensitive NAGK while arginine insensitive NAGK has no mobile N-terminal helix. The differences in their N-terminal segments appear to be highly correlated to their different subunit-subunit interactions and formation of different quaternary structures. The C-terminal has typical GNAT fold with a seven-stranded β sheet as its central core and three α helices hanging on both sides to form αβα sandwich structure. Superimposition of the NAT domain of mmNAGS/K and ngNAGS results in a root mean square deviation of 2.5 Å, with 112 aligned residues. Significant differences are found in the C-terminal arm. mNAGS/K has a much shorter loop to link β18 and β19 than ngNAGS, which has two extra helices (α14’ and α14) (Figure 2C). Instead, a long helix, α15, at the C-terminal of mmNAGS/K occupies the equivalent position of α14 in ngNAGS (Figure 2D). A 1–3 amino acid residue flexible linker fuses the two domains. The relative orientation between the two domains appears to be affected by inhibitor binding and the different packing environments in crystal structures, and appears to be closely related to the regulation mechanism [44,46,47].

## 5. Quaternary Structure of NAGS

**Figure 3:**
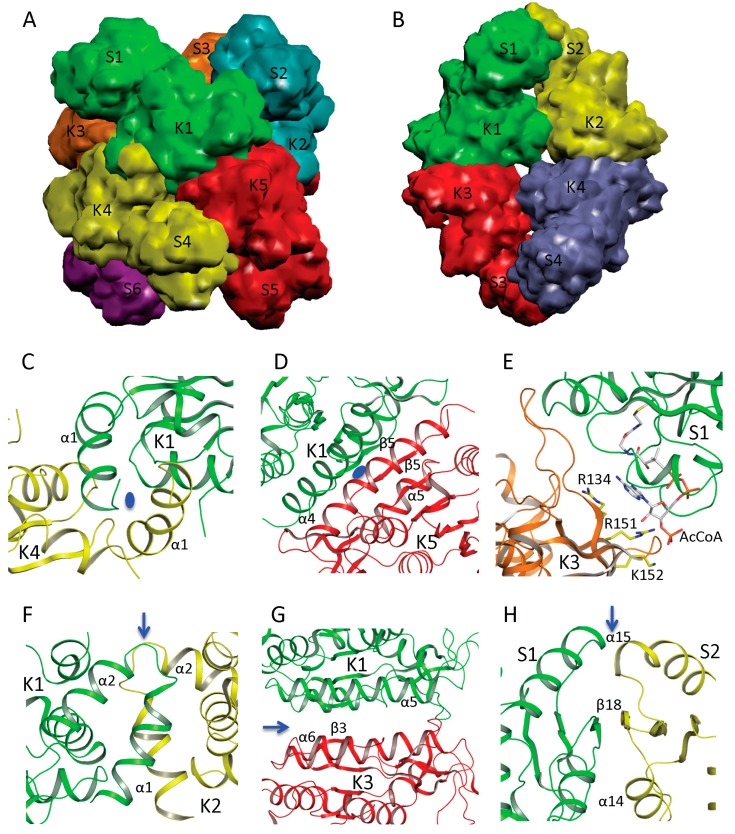
Quaternary structure and interface interactions of ngNAGS and mmNAGS/K. Simplified model of the hexamer architecture of ngNAGS (**A**) and the tetrameric architecture of mmNAGS/K (**B**). Ribbon diagram of three different types of interface for ngNAGS: K1–K4 interface (**C**), K1–K5 interface (**D**), and S1-K3 interface (**E**), in comparison to those of mmNAGS/K: K1–K2 interface (**F**), K1–K3 interface (**G**), and S1–S2 interface (**H**). K1–K4 and K1–K5 interfaces of ngNAGS have two-fold symmetry, whose axes, indicated by a blue oval, are perpendicular to the plane. As a result, in K1–K5 interface the central β5 interacts with the equivalent β5 from another subunit in an antiparallel fashion to form a continuous β strand across this interface. No symmetry exists for S1–K3 interface of ngNAGS, which will change upon arginine binding to enzyme. All three types of interfaces of mmNAGS/K have a two-fold symmetry, indicated by blue arrows, which are parallel to the plane. As a result, the central β3 positions with the equivalent β3 from other subunit in a parallel fashion in K1–K3 interface of mmNAGS/K, which is different from that in ngNAGS. The central β18 interacts with the equivalent β18 from another subunit antiparallel in S1–S2 interface of mmNAGS/K, forming a continuous β strand across the interface. No equivalent interface is found in ngNAGS.

In NAGS from *N. gonorrhoeae* (ngNAGS), six subunits assemble via the N-terminal AAK domains with D3 symmetry to form a toroidal shaped structure with three NAT domains on one side and three NAT domains on the other side of the hexamer (Figure 3A). Although this is the only member of the bacteria-like NAGS group with a determined structure, it is likely conserved among members of this group. paNAGS has also been shown to be a hexamer in solution [10]. In contrast to the bacteria-like NAGS configuration represented by ngNAGS, the NAGS structures of *M. maris* and *X. campestris* are tetramers in which the AAK domains of the subunits are in the middle of the molecule and the NAT domains are at both polar ends (Figure 3B). The whole molecule has a D2 symmetry, which is different from ngNAGS. Although the protein sequences are highly diverse [17], the tetrameric structure seems to be a common feature among the vertebrate-like group. Consistent with this finding, NAGK from *Saccharomyces cerevisiae*, has a sequence similar to the vertebrate-like group and similar two-domain structures, as well as similar tetrameric quaternary structures [48].

AAK domains nucleate the quaternary structures of both bacteria-like and vertebrate-like NAGS through two different interfaces. The first interface involves the N-terminal segment. In ngNAGS, this interface (K1–K4 interface) is formed by interlacing the N-terminal helices from adjacent subunits (Figure 3C). In mmNAGS/K, the two helices in the N-terminus interact in parallel to two other equivalent helices from the adjacent subunits, to form this interface (K1–K2 interface) (Figure 3F). The second interface (K1–K5 in ngNAGS and K1–K3 in mmNAGS/K) involves the N-terminal lobe of the AAK domain. This subunit surface is commonly used to form the dimer structures in all other amino acid kinases, including *E. coli* NAGK [49], *Pyrococcus furiosus* UMP kinase [50], and *E. coli* glutamate 5 kinase [51]. However, the detailed interactions involving this interface are very different. Even though the two subunits involved in this interface are related to each other by two-fold symmetry, in both ngNAGS and mmNAGS/K, the two-fold symmetry axis is perpendicular to the plane of the central β-strand in ngNAGS, while it is parallel to the central β-strand in mmNAGS/K. In comparison to the equivalent subunit surface in ngNAGS, the subunit surface in mmNAGS has relative rotation about 117° between subunits around an axis perpendicular to the interacting surface (Figure 3D,G).

In ngNAGS, no interaction between NAT domains from different subunits is found. Instead, the NAT domains interact with the AAK domains from the other subunits. This interaction (S1–K3 in ngNAGS) is weak but important for the regulation of NAGS activity (Figure 3E), because arginine binding changes this interface interaction completely. It is noteworthy that the structure of the C-terminal NAT domain without AAK domain from *Vibrio parahaemolyticus* (PDB code 3E0K) shows a domain swapped dimer. However, this dimer should not be biologically relevant because no such interactions were identified in the full-length structure in ngNAGS. In mmNAGS, the NAT domain interacts with the equivalent domain from adjacent subunit to form S1-S2 interface (Figure 3H). The pairing of NAT domains is involved in the antiparallel hydrogen bonding of the central β strands to form a continuing β-sheet, in addition to the hydrophobic interaction between helices from subunit 1 and 2. The dimer structure of NAT domains is maintained for the isolated NAT domain as well. The isolated C-terminal NAT domain from human and *Xylella fastidiosa* were recently shown to be a dimer in solution, and form the same dimer interface as the full-length mmNAGS/K in the crystal structures [52,53]. This situation differs from the C-terminal DUF619 domain of scNAGK, in which the full-length structure shows very similar interactions of the C-terminal DUF619 domain, but, the isolated domain remains a monomer [48].

## 6. Active Site and Catalytic Mechanism

Since the short NAGS without the N-terminal domain of NAGS displays an extremely high *K*_m_ for glutamate [10,24], it was speculated previously that an efficient glutamate binding site might be provided by the AAK domain [7,42]. It is now clear, however, that both substrates, AcCoA and glutamate, bind in the NAT domain. Even though the sequence similarity for the NAT domain in ngNAGS and mmNAGS is low (15% sequence identity), both AcCoA and glutamate bind in a similar location within the cleft of the V-shaped GNAT fold [44,45,52,53,54]. The unique characteristics of the CoA recognition motif, which conforms to the (Arg/Gln)-Xaa-Xaa-Gly-Xaa-(Gly/Ala), can be seen in all ngNAGS, mmNAGS/K and human NAGS. The AcCoA and CoA bound ngNAGS structures clearly demonstrate that the *S*-acetylpantetheine moiety of AcCoA forms a pseudo-antiparallel β-sheet interaction with β22, and the pyrophosphate moiety interacts with the CoA recognition motif (Figure 2C). Even though no AcCoA or CoA bound structures of vertebrate-like NAGS are available, probably due to weaker affinity for AcCoA compared to bacteria-like NAGS, it is believed that AcCoA binds to the enzyme in a way similar to its binding to ngNAGS and other Gcn5-related acetyltransferases.

**Figure 4:**
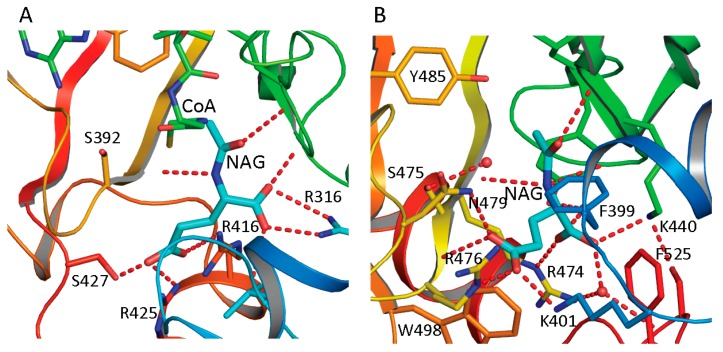
NAG binding site of ngNAGS (**A**) and hNAGS (**B**). The protein is shown in ribbon diagram. The bound NAG is shown as thick cyan sticks. The residues involved in binding NAG are shown as sticks. The hydrogen bonds are shown as red dashed lines. NAG binds to the protein in different conformations and using different sets of amino acid residues in ngNAGS and hNAGS.

The NAG bound structures of both ngNAGS and human NAGS enable the glutamate binding sites to be characterized in detail. A comparison of NAG bound structures of ngNAGS and hNAGS is shown in Figure 4A,B. The acetyl and the α-amino groups of NAG bind to the protein in a very similar way for both ngNAGS and hNAGS. However, the α-carboxyl and γ-carboxyl groups of NAG are in different locations and use different sets of amino acid residues to anchor to the protein. In ngNAGS, the side-chains of Arg316, Arg425 and Ser427, in addition to the main-chain N of Leu314 and Cys356, are involved in binding the two carboxyl groups. In hNAGS, the side-chains of Lys401, Arg474, Arg476 and Asn479 contribute hydrogen-bonding interactions for positioning the carboxyl groups. Two water-mediated interactions with the side-chain of Tyr441 and the main-chain N of Ser524 are also involved in binding the α carboxyl group. The NAG binding cavity of hNAGS appears to be larger than that of ngNAGS. The recently available structure of the NAT domain alone of xfNAGS/K bound with NAG demonstrate that NAG can bind to the protein in two different conformations, implying that the glutamate binding site for vertebrate-like NAGS might have more plasticity than its counterpart in bacteria-like NAGS [53].

The structures of ngNAGS bound with CoA and NAG, and with the bisubstrate-like analog, CoA-*S*-acetyl-l-Glu, suggest that the enzyme uses a direct attack mechanism to catalyze the reaction [45,54]. The similarities of the AcCoA and glutamate binding sites to those of ngNAGS imply that the vertebrate-like NAGS uses the same catalytic mechanism. In order for the α amino group of glutamate to attack the carbon atom of the acetyl group of AcCoA, a general base to facilitate the depronation of the α-amino group is usually needed. In ngNAGS, Glu353 was proposed to play this role [54]. In hNAGS, Tyr441 was proposed to play the similar role. The equivalent Tyr353 in mmNAG/K, Tyr361 in xcNAGS/K and Tyr352 in xfNAGS/K, can be identified as well [53]. After formation of the tetrahedral intermediate, a general acid to assist the protonation of the releasing CoA may also be required. In most of Gcn5 acetyltransferases, a tyrosine can be found to play this role including in vertebrate-like NAGS (Tyr485 in hNAGS and Tyr397 in mmNAGS/K) [52,53,55]. However, in ngNAGS, no such equivalent tyrosine residue is found, instead, the nearby Ser392 replaces it as a general acid [54].

## 7. Arginine Binding Site and Regulatory Mechanism

**Figure 5:**
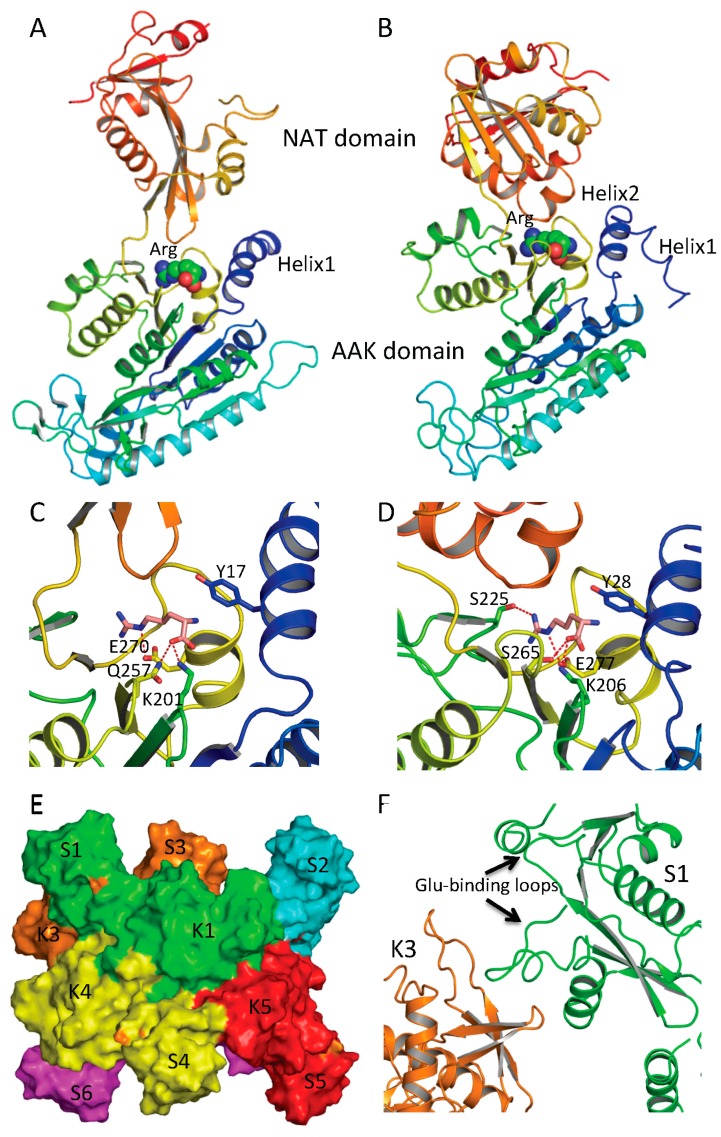
Arginine binding site and conformational changes. (**A**) Ribbon diagram of subunit structure of ngNAGS in the presence of arginine; (**B**) Ribbon diagram of subunit structure of mmNAGS/K in the presence of arginine. Arginine is shown in space filling model; (**C**) The details of arginine binding site of ngNAGS; (**D**) The details of arginine binding site of mmNAGS/K. The arginine and key residues involved in binding arginine are shown in sticks; (**E**) Simplified model of quaternary structure in the presence of arginine; and (**F**) The K3-S1 interface of ngNAGS in the presence of arginine.

Structural and mutagenesis studies show that the arginine-binding site of NAGS and arginine sensitive NAGK is conserved across phyla [42,46,47,56]. The arginine-binding site is located at the C-terminus of the AAK domain, close to the AAK and NAT domain interface and the C-terminal segment of the N-terminal helix of protein (helix 1 in ngNAGS, helix 2 in mmNAGS/K) (Figure 5A,B). The primary sequence has a conserved motif of E-(L/I)-(F/M)-(T/S)-X-X-G-X-G-T [56], which forms a loop connecting the last helix and the last β strand of the AAK domain. The arginine binds to the protein in such a way as to place the Cα on the N-terminal helix, the α-carboxyl group towards the central β-sheet and the side-chain to be encircled by the loop. A key lysine from the central β-sheet (K201 in ngNAGS and K206 in mmNAGS/K) and a key glutamate from the last helix of AAK domain (E270 in ngNAGS and E277 in mmNAGS/K) with many main-chain oxygen atoms help to position arginine in this position (Figure 5C,D).

The ngNAGS and mmNAGS/K structures with and without arginine bound provide significant insights into arginine induced regulation mechanisms [44,45,46,47]. In ngNAGS, arginine binding tightens its site and triggers the N-terminal helix movement to enhance K1–K5 interface interaction. As a result, the conformation of the whole hexamer changes significantly with a ~20 Å contraction along its three-fold axis and ~10 Å expansion for its hexamer ring (Figure 5E). The magnitudes of these changes are comparable with those found in arginine-sensitive NAGK structures [42], implying that arginine induced global conformational changes depend on the AAK domain only. As the hexamer ring widens, the linker between the AAK and NAT domains tightens, trigging a significant changes in the relative orientation of the NAT and AAK domains, re-establishing the K3-S1 interface interaction (Figure 5F). Eventually, the glutamate binding loops (helix10–helix11 and helix14-β25) become disordered and less able to bind the substrate. Mutagenesis and biochemical studies are consistent with this regulatory mechanism. The presence of arginine mainly affects the *K*_m_ for glutamate and the elongation of the linker between AAK and NAT domains completely abolishes the arginine inhibition [41]. In the absence of an AAK domain, as in the short NAGS from *Mycobacterium tuberculosis*, the *K*_m_ of glutamate can be as high as 600 mM [24].

mmNAGS/K and other vertebrate-like NAGS have tetrameric structures that are totally different from bacteria-like NAGS and the arginine regulation mechanism appears to be quite different. In the absence of arginine, the relative orientation between the AAK and NAT domains in vertebrate-like NAGS is quite flexible. The difference of the orientation angle can be as large as 29° between open and closed conformations [44]. In the presence of arginine, the subunit is in the closed conformation, which blocks AcCoA binding and therefore inhibits NAGS activity (Figure 5B) [47]. Interestingly, the relative orientation differences between AAK and NAT domains in the mature scNAGK structure for different subunits are up to 55°, reflecting similar conformational plasticity. Although the structure of mature hNAGS has not yet been determined, hNAGS and mNAGS both exist as tetramers in solution and pairing of the NAT domains is similar and thus it seems likely that the molecular mechanism of arginine activation is similar [52]. The respective domains are close only to a certain degree to promote catalytic activity, rather than to completely block the AcCoA binding site to inhibit it. The arginine titration curve of xcNAGS/K gave some clues for the mechanism. In the presence of very low concentration of arginine (<0.1 mM), arginine enhances NAGS activity about 20%, even though higher concentration of arginine inhibits NAGS activity completely by blocking the AcCoA binding site [17,52].

## 8. Evolution of Primary and Quaternary Structures

**Figure 6:**
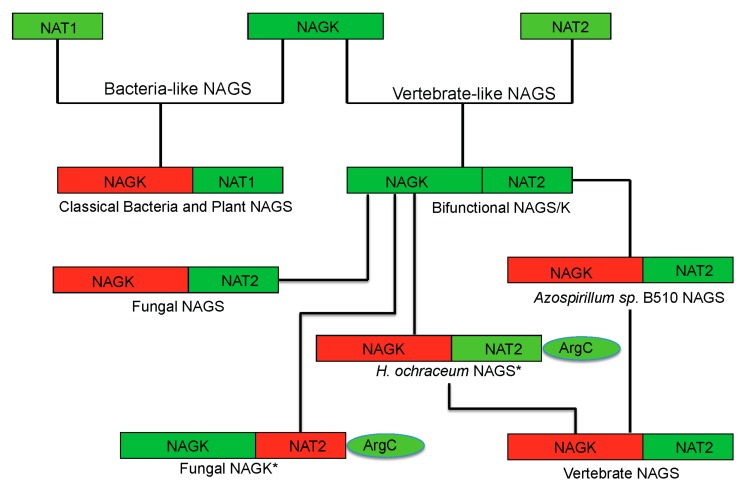
Proposed evolutionary path of the bacteria-like and vertebrate-like NAGS. The domains that have and have not enzymatic activity are colored green and red, respectively. The genes for fungal NAGK and *Haliangium ochraceum* NAGS (marked with *) encode a three-domain preprotein, whose mature proteins are formed likely by cleaving the argC domain.

Phylogenetic analysis has shown that classical bacteria and plant NAGS cluster in one group while bifunctional NAGS/K, fungal NAGK and NAGS, and vertebrate NAGS cluster in another large group [17]. The different quaternary structures of representative members of each group are consistent with the distant relationship between these two groups, implying that the separation of these two groups is a very early evolutionary event, as predicted previously [57]. However, the conservation of the arginine binding site and the general folding of the AAK domain indicate that the domains of both groups may be derived from the same common ancestor. The low sequence similarity and the non-conservation of the glutamate binding site in the NAT domains of the two groups demonstrate either that the NAT domains for different groups may come from different ancestors or that the separation of their NAT domains is likely a much earlier event before they fused with AAK domain to form NAGS. In the vertebrate-like NAGS group, the bifunctional NAGS/K appears to be the progenitor of other members positioned close to the root of the phylogenetic tree [17]. Interestingly, the bacteria whose genomes encode bifunctional NAGS/K also encode the gene for *N*-acetyl-l-ornithine transcarbamylase (AOTCase) in the arginine biosynthetic pathway, suggesting a coevolution of these two genes [17]. Phylogenetic analysis of the transcarbamylase family showed that the group of AOTCase clustering with *N*-succinyl-l-ornithine transcarbamylase [29] and *YgeW* encoded transcarbamylase of unknown function [58] is close to the root of the tree [59]. The primordial bifunctional NAGS/K evolved further to become the present day vertebrate NAGS, fungal NAGS and NAGK. The vertebrate NAGS and fungal NAGS preserved acetyltransferase activity, but lose the kinase activity while the fungal NAGK does the opposite. However, the present day fungal NAGK is formed by proteolytic processing of the biprotein precursor encoded by fusing an additional down-stream gene, *argC* [60,61]*.* Searches of the bacterial genomes reveal that the *NAGS* gene found in some bacteria appears to be the vestiges during this evolution. In the *Azospirillum* sp. B510 genome, the *NAGS* gene (AZL_c01370 is incorrectly annotated as *argC*, since *argC* encodes *N*-acetyl-γ-glutamyl-phosphate reductase), which has close sequence similarity with human (41.8%) and xcNAGS/K (76.7%), has lost its NAGK activity as a result of the elimination of key residues in the NAGK active site (R66D, N158G, K217T, *E. coli* NAGK numbering). Instead, a protein encoded by a different gene (AZL_005260) performs the NAGK function. In the *Haliangium ochraceum* genome, where there is a separate gene (Hoch_4935) for a bacterial-type NAGK, the gene for NAGS appears fused to the *argC* gene for *N*-acetyl-γ-glutamyl-phosphate reductase. Thus, this gene (Hoch_4300, incorrectly annotated as *argC* only) resembles the yeast gene encoding NAGK and *N*-acetyl-γ-glutamyl-phosphate reductase. Whether this protein in *H. ochraceum* undergoes proteolytic processing to cleave the argC domain as in yeast remains to be determined. The evolutionary paths of the typical NAGS are summarized in Figure 6.

## 9. Concluding Remarks

As a result of convergent evolution, many proteins have the capacity to catalyze the production of NAG from AcCoA and glutamate. Many more studies are needed to elucidate how these proteins bind substrates, whether they use the same catalytic mechanisms, where arginine binds and how arginine regulates their activity. The current knowledge about structure-function relationship is derived mainly from the two major groups of NAGS, bacteria-like and vertebrate-like. They are both two-domain proteins with an AAK domain at the N-terminus and a NAT domain at the C-terminus. Even though only the NAT domain has significant NAGS activity, the AAK domain provides the structural architecture for the enhancement of NAGS activity and for arginine binding and regulation. In mammalian counterparts, there is an extra N-terminal variable segment in addition to the AAK domain. This segment may be involved in protein-protein interactions with other mitochondrial protein partners [52]. Currently, little is known about the function and structure of H-NAGS. Whether argH plays a similar role to the AAK domain in the typical NAGS remains to be established. The possibility that the short version of NAGS (S-NAGS) and O-NAGS interact with other protein partners *in vivo* cannot be ruled out without further experiments. The recent discovery of a novel type of NAGS (C-NAGS) provides a new and fascinating opportunity to explore how different structures can catalyze the same reaction [25].
